# Tools for Screening, Predicting, and Evaluating Sepsis and Septic Shock: A Comprehensive Review

**DOI:** 10.7759/cureus.67137

**Published:** 2024-08-18

**Authors:** Kanishk Aggrawal, Sakshi Verma, Mason T Stoltzfus, Bhupinder Singh, FNU Anamika, Rohit Jain

**Affiliations:** 1 Internal Medicine, Dayanand Medical College and Hospital, Ludhiana, IND; 2 Internal Medicine, Government Medical College, Amritsar, Amritsar, IND; 3 Neurosurgery, Penn State College of Medicine, Hershey, USA; 4 Internal Medicine, Icahn School of Medicine at Mount Sinai, Queens Hospital Center, Queens, USA; 5 Medical School, University College of Medical Sciences, New Delhi, IND; 6 Internal Medicine, Penn State Health Milton S. Hershey Medical Center, Hershey, USA

**Keywords:** modified shock index, shock index, quick sequential organ failure assessment (qsofa), sequential organ failure assessment (sofa), septic shock (ss), sepsis

## Abstract

Sepsis is characterized by life-threatening organ dysfunction due to dysregulated host response to infection. It can progress to cause circulatory and cellular/metabolic abnormalities, resulting in septic shock that may significantly increase mortality. The pathophysiology of sepsis involves a complex interplay of invading pathogens and the body’s immune defense, causing alteration in normal homeostasis, eventually leading to derangements in the cellular, humoral, circulatory, and metabolic functions. Several scoring systems have been developed to rapidly predict or suspect sepsis, such as Sequential Organ Failure Assessment (SOFA), modified SOFA (mSOFA), quick SOFA (qSOFA), shock index (SI), and modified SI (mSI). Each of these scores has been utilized for triaging patients with sepsis, and as per medical advancements these scoring systems have been modified to include or exclude certain criteria to improve their clinical utility. This review aims to compare the individual scores and their usage for sepsis that may be used for laying the foundation for early recognition and prediction of sepsis and for formulating more precise definitions in the future.

## Introduction and background

The World Health Organization has declared sepsis a global health priority pertaining to an alarming increase in the incidence despite advancements in technological and therapeutic management, with a high risk of in-hospital mortality accounting for up to 20% of all-cause deaths worldwide [1,2]. The concerning rise in the incidence of sepsis and sepsis-related mortality is attributed to several reasons such as the advanced average age of patients, particularly in Western countries, the increase in invasive procedures, and widespread antibiotic resistance [3]. Sepsis is defined as “life-threatening organ dysfunction caused by a dysregulated host response to infection.” Whereas septic shock is defined as a condition in which sepsis causes circulatory and cellular/metabolic abnormalities that may significantly increase mortality. According to the recent third international consensus for sepsis and septic shock, the clinical criteria for septic shock includes sepsis and the need for vasopressor therapy to increase mean arterial pressure (MAP) ≥65 mmHg and lactate >18 mg/dL after resuscitating with adequate fluid [4]. The definition of sepsis also comprises clinical criteria based on the Sequential Organ Failure Assessment (SOFA) score, which includes a suspected or documented infection and an acute increase of ≥2 SOFA points [4,5]. The SOFA score includes parameters of six organ systems (respiratory, cardiovascular, neurological, hepatic, hematological, and renal). Each system is scored from 0 to 4 which is added to obtain a total score ranging from 0 to 24. SOFA can be easily calculated at the bedside and does not require the use of any computer software. However, the SOFA has been modified to include SpO_2_ instead of PaO_2_ and SpO_2_/FiO_2_ ratio in place of PaO_2_/FiO_2_ as a better alternative. The changes in modified SOFA (mSOFA) variables are found to have same-to-better accuracy in comparison to the conventional SOFA score [6]. Seyed Abdollah Emadi et al. have suggested the use of mSOFA over SOFA to predict mortality in patients with sepsis as it is easier to use [7]. For quick identification of patients with infections, a quick SOFA (qSOFA) score is introduced, which consists of three components, including systolic blood pressure (SBP) <100 mmHg, altered mental status (Glasgow Coma Scale (GCS) <15), and respiration rate (RR) ≥22 breaths per minute. In patients outside of the intensive care unit (ICU), a qSOFA score of ≥2 is strongly predictive of higher all-cause mortality [8]. The shock index (SI) is another screening tool that is calculated by dividing heart rate (HR) with SBP. Previously, SI has been used to aid identification of multiple shock conditions, particularly in trauma patients [9]. It was found that SI correlated well with the patient’s need for hospital/ICU stay, ventilation support, and blood transfusions. Compared to HR and blood pressure alone, SI is thought to be a more accurate measure for determining the severity of shock. However, one limitation of SI is that it completely excludes diastolic blood pressure (DBP), which in a retrospective study has been shown to be a good indicator of the severity of shock [10]. There have also been concerns regarding its use and appropriateness for all age groups, particularly for older patients [11]. To address these concerns the SI has been modified which can be calculated by dividing the HR with MAP. Hence, the modified shock index (mSI) is the function of stroke volume and systemic vascular resistance and has the potential to be a comprehensive assessment tool as it considers important information about cardiovascular and hemodynamic stability by incorporating HR, SBP, and DBP [12]. Hence, this review aims to highlight the importance and utility of these tools in the screening, prediction, and evaluation of sepsis and septic shock. We hope to provide detailed information by making a comparison among all these tools that may be used for laying the foundation for early recognition and prediction of sepsis and for formulating a more precise definition of sepsis in the future (Table 1).

**Table 1:** Tools for evaluating sepsis. This table outlines various tools that can be used to evaluate sepsis. SOFA: Sequential Organ Failure Assessment; mSOFA: modified Sequential Organ Failure Assessment; qSOFA: quick Sequential Organ Failure Assessment; MAP: mean arterial pressure; CNS: central nervous system; GCS: Glasgow Coma Scale score; HR: heart rate; SBP: systolic blood pressure; AMS: altered mental status

Criteria	SOFA	mSOFA	qSOFA	Shock Index	Modified Shock Indes
Respiratory	PaO_2_/FiO_2_ ≤400	SpO_2_/FiO_2_ ≤400	-	-	-
Coagulation	Platelets <150 × 10^3^	Platelets <100 × 10^3^	-	-	-
Liver	Bilirubin >1.2	-	-	-	-
Cardiovascular	MAP <70 mmHg	MAP <70 mmHg	-	-	-
CNS	GCS ≤13	GCS ≤13	-	-	-
Renal	Creatine >1.2	-	-	-	-
Shock Index	-	-	-	HR/SBP >0.7	HR/MAP ≥1.0
qSOFA	-	-	Respiratory rate ≥22/minute, AMS, SBP ≤100 mmHg	-	-

## Review

Updated pathophysiology of sepsis

The pathophysiology of sepsis is complex and driven by mechanisms involving pathogenic antigens and complementary responses of the immune system. The interplay of invading pathogens and the host’s immune defense produces alteration in normal homeostasis leading to derangements in the cellular, humoral, circulatory, and metabolic functions [13].

Infection

Infection is the initial stage of septic shock and triggers multifaceted and prolonged host response, involving both proinflammatory and anti-inflammatory mechanisms, which can play a role in either eliminating the infection and promoting tissue healing or causing harm to organs and potentially leading to additional infections. The particular response in each patient is influenced by the type of pathogen (load and virulence) and the individual’s genetic traits and concurrent health conditions, with variable responses at the local, regional, and systemic levels [14].

Cell-Mediated Innate and Adaptive Immune Responses

The innate immune system includes a host of cells and proteins, including neutrophils, natural killer cells, complement proteins, macrophages, and others. It directly destroys pathogens and activates the adaptive immune system for more specific pathogen destruction and immune system regulation. The innate system detects pathogens and injury through toll-like receptors (TLRs) which recognize carbohydrate and lipid molecules commonly found on the surface of pathogens known as pathogen-associated molecular patterns (PAMPs) [15]. Additionally, they recognize damage-associated molecular patterns (DAMPs), which are molecules released from damaged host cells, including mitochondrial DNA, ATP, and high mobility group box 1 (HMGB1) [16]. DAMPs and PAMPs interact with pattern recognition receptors on the cell surface (such as toll-like receptors and C-type lectin receptors) or within cells (such as NOD-like receptors and RIG-I-like receptors). This interaction triggers complex metabolic cascades responsible for the formation of the inflammasome [16,17]. The inflammasome leads to the synthesis and release of pro-inflammatory cytokines such as interleukin-1-beta (IL-1ß), tumor necrosis factor-alpha (TNF-α), interleukin-6 (IL-6), interleukin-18 (IL-18), and HMGB-1. In some cases, an exacerbated inflammasome activity can result in a cytokine release syndrome or “cytokine storm” [17]. In the adaptive immune system, when CD4+ T cells become activated, they differentiate into specific T-helper (Th) subsets, including Th1, Th2, and Th17. Th1 cells play an important role in expanding memory T cells by secreting IL-2, initiating the activation of CD8+ T cells, and producing the pro-inflammatory cytokine interferon-gamma (IFN-γ), which enhances the phagocytosis and elimination of microbes. Th2 cells release IL-4 and IL-5, which promote the switching of B lymphocytes, and IL-10 to resolve inflammation [18]. Maintaining a balance in the communication between Th1 and Th2 is essential for effectively clearing infection, but when this balance is disrupted, as seen in conditions with sepsis, it can lead to autoimmune diseases, secondary infections, reactivation of viruses, long-term immunosuppression, immune collapse, and even physical disabilities, also known as persistent inflammation immune-suppression catabolism syndrome [18,19]. Sepsis-induced immunosuppression is characterized by the release of anti-inflammatory cytokines, the death of immunocytes, exhaustion of T cells, and excessive production of immunomodulatory cells, including myeloid-derived suppressor cells (MDSCs) and regulatory T cells (Tregs). This condition is associated with a reduction in the expression of human leukocyte antigen-DR (HLA-DR) and an increase in the expression of immune checkpoint molecules (such as T-cell immunoglobulin, programmed cell death 1 (PD-1) and mucin domain-containing protein-3 (TIM-3), and B and T lymphocyte attenuator (BTLA)). These factors further exacerbate immunosuppression, ultimately leading to secondary infections and multiple organ dysfunction syndrome (MODS), which is the primary cause of poor prognosis in septic patients [19].

Endothelial Dysfunction

Endothelial cells play a crucial role in the systemic response following bacterial infection by limiting its dissemination and endothelial damage results in phenotypic and physical changes in the endothelium, affecting the release of vasodilators such as nitric oxide and prostacyclin, reducing reactivity to vasoconstrictors, promoting the aggregation of white blood cells and platelets, and increasing the expression of inducible nitric oxide synthase which affects nitric oxide signaling [20-22]. Sepsis activates circulating neutrophils and their recruitment to release mediators such as reactive oxygen species, proteases, prostaglandins, coagulation activation, and coagulation-derived proteases [21]. Additionally, damage to glycocalyx and vascular tone dysfunction disrupt microcirculatory blood flow, causing organ damage and, potentially, life-threatening organ failure [20]. The glycocalyx plays a critical role in maintaining hemostasis, vascular barrier function, leukocyte, and platelet adhesion, transmitting shear stress to the endothelium, and anti-inflammatory and antioxidant defenses. Reactive oxygen species such as hydrogen peroxide, hydroxyl anions, and superoxide are believed to be the main factors causing the shedding of the glycocalyx but other mediators include TNF-α and heparanase. This loss-of-barrier function leads to edema and is a significant contributor to organ failure in sepsis [23]. The glycocalyx in renal glomerular capillary endothelial cells and lungs appears to be particularly susceptible to degradation in sepsis [24].

Metabolic Derangement

In septic patients, mitochondrial dysfunction is caused by several mechanisms, including reversible electron transport chain complex inhibition, cytochrome c oxidase inhibition, respiratory uncoupling, oxidative inhibition of mitochondrial dehydrogenases and adenine nucleotide transporters, and decreased cytochrome content [25]. This overall reduction in cellular energy expenditure is consistent with retained tissue oxygen tension in sepsis, and this reduction contributes to organ dysfunction as many cells reduce specialized functions. This can contribute to or exacerbate problems such as liver dysfunction, acute kidney injury, myocardial depression, encephalopathy, acute lung injury, and decreased barrier and transport functions in the gastrointestinal tract [16].

In sepsis, the metabolism of all macronutrients is altered. Glycolysis increases and the inability to channel pyruvate into the tricarboxylic acid cycle results in increased lactate production. Sepsis also increases lipolysis, increasing levels of fatty acids and triglycerides; however, the utilization of these substances is disrupted, which can lead to the buildup of lipids and their toxic byproducts [26].

Coagulopathy

The dysregulation of blood clotting in sepsis involves two important components of the innate immune system: platelets and neutrophils. Activated platelets express P-selectin which facilitates interactions with leukocytes through the P-selectin glycoprotein ligand-1. Platelets bind to neutrophils through the platelet toll-like receptor 4 (TLR4) that stimulates the production of neutrophil extracellular traps (NETs). Although NETs protect the host by limiting microbial growth and dissemination, excessive NET production during sepsis can shift the balance toward excessive coagulation, promoting thrombus formation; moreover, NETs also promote the stability of fibrin clots and inhibit plasminogen activators [27]. Damage to the endothelium by bacterial toxins leads to the upregulation of tissue factor, which, in turn, activates factor VII, initiating the extrinsic coagulation pathway. The generation of thrombin also leads to activation of platelets and, conversely, activated platelets contribute to the generation of thrombin by releasing procoagulant factors when they degranulate [28].

Tools for evaluating sepsis

Sepsis is a clinical syndrome defined by multiorgan failure due to a maladaptive host system response to various infections. In contrast, septic shock is a congregation of signs and symptoms often associated with cardiovascular dysfunction with characteristic lab abnormalities such as hyperlactatemia and persistent hypotension despite adequate fluid resuscitation [29,30]. Patients suffering from sepsis often present with a broad spectrum of symptoms such as general fatigue and non-specific symptoms, including fever, tachycardia, tachypnea, confusion, difficulty breathing, or reduced urine output, with some patients experiencing skin mottling with increased capillary refill time. In addition to varied symptoms, increased lactate levels and white blood cell count with increased plasma C-reactive protein or procalcitonin levels often aid in diagnosing sepsis and septic shock [30]. Due to absurd and vague symptoms and laboratory parameters, various tools such as SI, MSI, SOFA, qSOFA, and mSOFA have been employed to enhance the triaging procedure and risk stratification and help identify critically sick patients earlier and more quickly to improve the assessment of febrile patients as early goal-directed therapy in the treatment of sepsis often has good prognostic implications for patients experiencing sepsis and septic shock [31].

SI is a valuable tool for the early recognition and evaluation of critical illness in the emergency department (ED), as well as for monitoring resuscitation progress. In a study conducted by Rady et al., an SI of 0.9 or higher was found to predict a higher priority for treatment in the ED, as well as a higher likelihood of hospital admission and intensive therapy upon admission, when compared to relying solely on pulse or blood pressure measurements [32]. Another retrospective cohort study included 58,336 adults in an ED and found that SI values between 0.5 and 0.7 (average) had the lowest likelihood of admission and inpatient mortality. In contrast, SI >1.2 conferred nearly 12 times more likelihood of being admitted than standard SI. In another study involving 295 patients with severe sepsis, it was found that 38.6% of patients with a sustained elevation in SI >0.8 for at least 80% of ED vital sign measurements required vasopressors within 72 hours of admission compared to only 11.6% of patients without a sustained elevation in SI [33,34]. SI has also been evaluated in predicting hemodynamic response to volume expansion, as shown by a prospective observational study of 25 patients, which showed that patients with a central venous pressure (CVP) ≥8 mmHg and SI ≤1 were unlikely to respond to volume expansion (13 non-responders and one responder), with a negative predictive value (NPV) of 93% (95% confidence interval (CI) = 71-100%). In contrast, patients with an SI >1 were more likely to be fluid-responsive, indicating the combination of a high CVP and relatively low SI is better than either alone when assessing if a patient will respond to further fluid boluses, which may aid in avoiding fluid overload in critically ill patients [35]. Another study by Berger et al. found SI ≥0.7 as effective as SIRS in NPV and the most sensitive screening test for hyperlactatemia and 28-day mortality. SI ≥1.0 was the most specific predictor of both outcomes [32]. In addition, a study by Romain Jouffroy and his team retrospectively analyzed 114 patients with septic shock, cared for by a medical ICU from 2016 to 2019. The study found that an SI >0.9, a readily available tool, can predict increased mortality risk for such patients initially cared for in the prehospital setting [36].

A modified version of the SI has been developed called the mSI that incorporates HR, SBP, and DBP as it is calculated by dividing the HR with MAP, providing a more comprehensive assessment that considers important information regarding stroke volume and systemic vascular resistance [10,37]. MAP is a crucial indicator for fluid resuscitation and vasopressor titration in patients with septic shock. It considers cardiac output, systemic vascular resistance, and SBP/DBP. In a retrospective cohort study by Jayaprakash et al., 578 patients were analyzed, and the results showed that an mSI >1.3 was linked to a higher risk of myocardial dysfunction (odds ratio (OR) = 1.10, 95% CI = 1.00-1.21; p = 0.058) and depression (OR = 1.28, 95% CI = 1.07-1.53; p = 0.007). The study also found associations between mSI >1.3 and ICU mortality (OR = 1.17, 95% CI = 1.04-1.32; p = 0.011), hospital mortality (OR = 1.13, 95% CI = 1.02-1.25; p = 0.025), and SOFA score [38]. Similarly, Althunayyan et al. conducted a retrospective cohort study of 274 febrile patients and found that mSI ≥1 was sensitive in predicting ICU admission (85%), shock (90%), and mortality (100%). On the other hand, mSI ≥1.3 was linked to sepsis, hyperlactemia, ICU admission, and 28-day mortality with a specificity range of 59-100% [31]. Furthermore, another study comparing mSI with primary outcomes in patients with acute coronary syndrome (ACS) found that a high mSI (≥1) was substantially linked with in-hospital mortality compared to a low mSI (<1) (OR = 4.36 (3.09-6.14), p < 0.001). The study also revealed that mSI moderately predicted in-hospital mortality in patients with ACS. After adjusting for possible confounders, the multivariate analysis showed that greater mSI (≥1) significantly correlated with in-hospital mortality (OR = 2.64 (1.67-4.20), p < 0.001) [39]. A study conducted by Zhang et al. analyzed the medical records of 1,266 patients with septic shock who required vasopressors. The study aimed to explore the correlation between SI, mSI, diastolic SI (dSI), and mortality rates within three days and throughout the hospital stay. The study revealed that 8.7% of the patients died within three days, and 23.5% of the patients died during their hospital stay. The multivariable logistic regression analysis found that pre-vasopressor SI/mSI/dSI had a significant association with three-day mortality in patients with septic shock who required vasopressors with fully adjusted models (Ps for trend < 0.01). The study also found that the area under the curve (AUC) of pre-vasopressor SI, mSI, and dSI were 0.746, 0.710, and 0.732 for three-day mortality, respectively. Additionally, there were significant differences in the time course of SI, mSI, and dSI between survivors and non-survivors at three-day/in-hospital mortality among patients with septic shock who required vasopressors [40].

The SOFA score is another tool developed to assess the acute morbidity of critical illness at a population level and has been widely validated in various healthcare settings and environments. A change in the SOFA score of 2 or more is now a defining characteristic of sepsis syndrome [41]. According to a study conducted by Innocenti et al., the SOFA score was significantly higher in septic patients who did not require intubation but had an adverse outcome regarding 28-day mortality and need for ICU admission [42]. A retrospective study by Khwannimit et al. analyzed 1,589 consecutive sepsis patients admitted to an ICU between January 2011 and December 2017 and found that the SOFA score was the accurate predictor of hospital mortality, with an area under the receiver operating characteristic curve (AUC) of 0.880. Additionally, the study found that the SOFA score helped predict 30-day mortality and multiple organ failures among sepsis patients admitted to the ICU [43]. A study conducted by Peng et al. demonstrated that the SOFA criteria are a quick and accurate way to screen patients at high risk for sepsis after percutaneous nephrolithotomy. The study included 31 patients, of whom 12 (2.7%) developed septic shock. The SOFA criteria had the highest sensitivity (100%) and greater specificity (87% vs. 81%) for identifying at-risk patients. The AUC of SOFA (0.973) was greater than that of qSOFA (0.928) and SIRS (0.935) in predicting septic shock. When combined with SIRS, SOFA outperformed qSOFA in discriminating septic shock (AUC = 0.987 vs. 0.978). According to the decision curve analysis, SOFA was superior to qSOFA and SIRS, with the highest benefit to septic patients [44].

The qSOFA score is a quick way to identify patients with infections and patients with sepsis and septic shock comprising three components, including SBP <100 mmHg, altered mental status (GCS <15), and RR ≥22 breaths per minute with a score of ≥2 in patients strongly associated with higher all-cause mortality. However, the role of qSOFA score in diagnosing sepsis patients in the ED showed that although the test had near-acceptable specificity, the sensitivity was relatively low, as shown by Wani et al. that out of 120 patients who had a positive qSOFA score, only 30 were later confirmed as having sepsis. In contrast, in the qSOFA-negative group, 14 patients were subsequently diagnosed with sepsis. Furthermore, the qSOFA score has poor sensitivity and specificity for predicting 28-day mortality. As in this study, the qSOFA score could successfully predict mortality in only 17 patients and failed to predict mortality in nine out of 26 deaths. Therefore, this suggests that the qSOFA score might not be a good screening tool for early detection of sepsis patients in the ED [45]. Another meta-analysis by Maitra et al. with 406,802 patients from 45 observational studies found that qSOFA ≥2 was poorly sensitive in predicting in-hospital mortality for hospitalized patients with suspected infection [46].

In contrast, a study by Baig et al. compared the effectiveness of qSOFA with SOFA scores in predicting in-hospital mortality among severe sepsis and septic shock patients in a low-middle-income country’s tertiary care hospital ED including 760 subjects, found that qSOFA’s area under the receiver operating curve (AUROC) of qSOFA for predicting mortality in subjects was 0.92 (95% CI = 0.89-0.94) with 96% sensitivity and 87% specificity in comparison to the AUROC of SOFA score which was 0.63 (95% CI = 0.55-0.70) with 71% sensitivity and 57% specificity, thereby showing that qSOFA is a valuable tool for predicting in-hospital mortality in severe sepsis and septic shock patients in a tertiary care hospital ED of a low-middle-income country [47].

Application of the SOFA and qSOFA has led to a decrease in ICU mortality rates for sepsis patients over the years. As populations age, sepsis is becoming more common among older patients with multiple health problems, including cancer and frailty. At least half of all patients with sepsis are treated outside of the ICU [48]. Identifying and diagnosing sepsis in EDs presents challenges, including distinguishing infections from non-infectious inflammatory conditions, delays in recognizing organ dysfunction, and distinguishing between acute and chronic organ dysfunction. To address these challenges in diagnosing sepsis, a modified version of the SOFA score, called the mSOFA score, has been developed, which uses more easily accessible substitutes for cardiac and respiratory parameters, thus making it practical for use in EDs [6]. According to a study conducted by Raymond et al. among 474 patients with probable sepsis found that the mSOFA score was a significant predictor of mortality at all the tested time points. Patients with a positive mSOFA score had a 25% mortality rate (22 out of 88), which was significantly higher than the 2.1% mortality rate (3 out of 140) of those with a negative mSOFA score (OR = 15.2, 95% CI = 4.4, 52.7; p < 0.001). The negative predictive value of the mSOFA score was 97.9% (95% exact CI = 93.9-99.6%), which could help identify patients with a low mortality risk. The study concluded that the mSOFA score predicts mortality when patients are admitted to the ED [6]. A study conducted by Grissom et al. showed that the SOFA and mSOFA scores, calculated on day 1, were equally effective in predicting mortality with an AUC of 0.83 (95% CI = 0.81-0.85) and 0.84 (95% CI = 0.82-0.85), respectively. Based on the study, it was concluded that the mSOFA score is as effective as SOFA in predicting mortality and is more straightforward to implement in settings with limited resources. However, using either score as a triage tool would exclude many patients who could otherwise survive [49]. Similar results were shown by Emadi et al. as mSOFA may be effective in predicting mortality similar to SOFA among patients, with its ease of use as it involves only a single lab parameter [7].

Sepsis scores, such as the SOFA and the qSOFA, are valuable tools for assessing the severity of sepsis and predicting outcomes but they often have several limitations that underscore the need to monitor specific organs and body functions. These scoring systems often are non-specific as they are designed to assess overall severity and prognosis, but they do not pinpoint specific organ dysfunctions such as cardiovascular or pulmonary dysfunction which often causes delays in identifying and treating the precise organ systems. In addition, these sepsis scoring systems often provide a snapshot of the patient’s condition at a single point in time and fail to capture rapid changes in a patient’s status, necessitating continuous monitoring and reassessment [50].

Given these limitations, it is crucial to monitor specific organs and body functions in addition to the use of sepsis scores which can provide more detailed information and help guide the treatment. For example, continuous monitoring of blood pressure, HR, and cardiac output is essential to detect and manage shock and other cardiovascular complications of sepsis. Similarly monitoring oxygen saturation, RR, and arterial blood gases helps manage respiratory failure and optimize ventilatory support. Moreover, regular assessment of urine output, serum creatinine, and blood urea nitrogen levels is crucial for detecting and managing acute kidney injury and monitoring liver function tests, including bilirubin, alanine aminotransferase, and aspartate aminotransferase can identify hepatic dysfunction early. In summary, while sepsis scores are useful for initial assessment and prognostication, they should be complemented with continuous and specific monitoring of organ functions. This integrated approach enables timely and targeted interventions, improving patient outcomes in sepsis management.

The Society of Critical Care Medicine (SCCM) derived and approved the Phoenix Sepsis Criteria using the data from several international databases, meta-analyses, and systematic reviews. The criteria assess the dysfunction of cardiovascular, respiratory, coagulation, and neurological systems by giving a score of 0-6, 0-3, 0-2, and 0-2, respectively. A Phoenix score of at least 2 can identify potentially life-threatening organ dysfunction in children below the age of 18 years [50]. According to the data by SCCM, the in-hospital mortality rate for children with a Phoenix Sepsis Score of at least two points was 7.1% in higher-resource settings and 28.5% in lower-resource settings. This is more than eight times greater than the rate for children with suspected infections who did not fulfill these criteria. Children with organ dysfunction in any one of the four systems, i.e., respiratory, cardiovascular, coagulation, or neurological, that was not the main site of infection had a greater death rate. Children with sepsis who exhibited cardiovascular dysfunction, defined as at least one cardiovascular point in the Phoenix Sepsis Score, such as severe hypotension relative to age, blood lactate levels higher than 5 mmol/L, or the requirement for vasoactive medication were considered to be in septic shock [51]. In higher and lower-resource environments, the in-hospital mortality rates for children suffering from septic shock were 10.8% and 33.5%, respectively. Sanchez-Pinto et al. conducted a retrospective cohort study in different healthcare systems in the United States, Colombia, Bangladesh, China, and Kenya to identify the external validity of the Phoenix criteria. Compared to the International Pediatric Sepsis Consensus Conference criteria, the Phoenix sepsis criteria performed better in the identification of pediatric sepsis and septic shock [52].

## Conclusions

The different scoring systems used in screening and identifying sepsis have their own advantages and limitations based on their sensitivity, specificity, and utilization in different settings. mSOFA can be compared to SOFA in predicting mortality; however, mSOFA is a better tool to use in settings with limited resources. Likewise, qSOFA has also been used as a quick tool for predicting mortality but it may not be a good tool due to its low sensitivity and specificity. On the other hand, mSI is superior to SI as it provides more comprehensive information. The new Phoenix score has been approved to identify sepsis in the pediatric population and has been shown to exhibit a higher positive predictive value.
